# A Clinically Relevant Variant of the Human Hydrogen Sulfide-Synthesizing Enzyme Cystathionine *β*-Synthase: Increased CO Reactivity as a Novel Molecular Mechanism of Pathogenicity?

**DOI:** 10.1155/2017/8940321

**Published:** 2017-03-22

**Authors:** João B. Vicente, Henrique G. Colaço, Francesca Malagrinò, Paulo E. Santo, André Gutierres, Tiago M. Bandeiras, Paula Leandro, José A. Brito, Alessandro Giuffrè

**Affiliations:** ^1^Instituto de Tecnologia Química e Biológica António Xavier, Universidade Nova de Lisboa, Oeiras, Portugal; ^2^Instituto Gulbenkian da Ciência, Oeiras, Portugal; ^3^CNR Institute of Molecular Biology and Pathology, Rome, Italy; ^4^Department of Biochemical Sciences, Sapienza University of Rome, Rome, Italy; ^5^Instituto de Biologia Experimental e Tecnológica, Oeiras, Portugal; ^6^Research Institute for Medicines and Department of Biochemistry and Human Biology, Faculty of Pharmacy, University of Lisbon, Lisbon, Portugal

## Abstract

The human disease classical homocystinuria results from mutations in the gene encoding the pyridoxal 5′-phosphate- (PLP-) dependent cystathionine *β*-synthase (CBS), a key enzyme in the transsulfuration pathway that controls homocysteine levels, and is a major source of the signaling molecule hydrogen sulfide (H_2_S). CBS activity, contributing to cellular redox homeostasis, is positively regulated by S-adenosyl-L-methionine (AdoMet) but fully inhibited upon CO or NO• binding to a noncatalytic heme moiety. Despite extensive studies, the molecular basis of several pathogenic* CBS* mutations is not yet fully understood. Here we found that the ferrous heme of the reportedly mild p.P49L CBS variant has altered spectral properties and markedly increased affinity for CO, making the protein much more prone than wild type (WT) CBS to inactivation at physiological CO levels. The higher CO affinity could result from the slightly higher flexibility in the heme surroundings revealed by solving at 2.80-Å resolution the crystallographic structure of a truncated p.P49L. Additionally, we report that p.P49L displays impaired H_2_S-generating activity, fully rescued by PLP supplementation along the purification, despite a minor responsiveness to AdoMet. Altogether, the results highlight how increased propensity to CO inactivation of an otherwise WT-like variant may represent a novel pathogenic mechanism in classical homocystinuria.

## 1. Introduction

Hydrogen sulfide (H_2_S) has emerged as a key signaling molecule in human physiology and pathophysiology, being implicated in the regulation of several processes such as neuromodulation, angiogenesis, vasorelaxation, bioenergetics/respiration, cell survival, and proliferation [1–4]. The gas has a pivotal role in the control of cellular redox homeostasis and prevention of oxidative stress, modulating the expression of key antioxidant enzymes [2]. Similarly to other relevant gaseous signaling molecules like CO and NO•, at low concentrations H_2_S can exert cytoprotective effects or become cytotoxic at higher concentrations.

At least three human enzymes have been identified as key endogenous sources of H_2_S: cystathionine *β*-synthase (CBS) and cystathionine *γ*-lyase (CSE), both occurring in the transsulfuration pathway of methionine metabolism and mercaptopyruvate sulfurtransferase (MST) [1]. Beyond enabling conversion of homocysteine to cysteine through their historically recognized canonical activities, CBS and CSE catalyze a number of “alternative” reactions leading to H_2_S synthesis, which has brought these enzymes into the limelight [1, 5, 6]. Indeed, a growing number of human pathologies, from cardiovascular and neurodegenerative diseases to different cancer types, are reportedly associated with disturbances of H_2_S metabolism related to CBS, CSE, and/or MST [7]. CBS, in particular, has been shown to be overexpressed in colorectal, ovarian, and breast cancer, among other cancer types (reviewed in [8, 9]), as well as in neurodegenerative diseases, such as amyotrophic lateral sclerosis [10]. The enzyme is therefore currently recognized as a drug target [8].

CBS catalyzes the condensation of homocysteine and serine (or cysteine) leading to formation of cystathionine and H_2_O (or H_2_S). The human enzyme is a 551-amino acid protein with a central catalytic domain, harboring a pyridoxal 5′-phosphate (PLP) cofactor, flanked by a C-terminal domain with a binding site for the allosteric positive regulator s-adenosyl-l-methionine (AdoMet) and an N-terminal domain, harboring a hexacoordinate heme with C52 and H65 as endogenous Fe ligands [11]. Structural studies have shown that whereas the AdoMet-binding domain occludes the substrate entry site in the catalytic core, AdoMet binding induces a conformational change clearing the path for substrates to access the active site [11–13]. In the presence of AdoMet enzymatic activity thus increases 2–5-fold, as measured with isolated proteins and bacterial or human cell lysates. Another interesting regulatory mechanism concerns the B-type heme moiety in the N-terminal domain. While the enzyme is fully active when the heme is in the oxidized state, reduction to the ferrous state negatively impacts enzyme activity, possibly through a ligand exchange mechanism involving the replacement of C52 by a yet unknown neutral ligand [14, 15]. Such change in the Fe coordination is accompanied by a notable shift in the CBS heme Soret band from 449 to 424 nm, leading to an inactive protein species commonly referred to as “C-424” [15]. Even more striking is that binding of NO• or CO to the ferrous heme results in enzyme inhibition [16–20], with different lines of evidence pointing to a physiological role of this regulatory mechanism in vivo [21–27]. According to structural and mutagenesis studies, changes in the heme redox and ligation state are communicated to the PLP active site through *α*-helix 8 [15, 28, 29]. This regulatory mechanism places CBS at the crossroad between the signaling pathways of the three gasotransmitters (H_2_S, CO, and NO•) in human physiology [30]. More recently, it has been shown that AdoMet enhances CBS sensitivity to CO and NO•, further highlighting an intricate interplay between the three domains in the protein [20].

Classical homocystinuria (OMIM #236200) is an inborn error of metabolism associated with mutations in the* CBS* gene. With a variable incidence of 1 : 1,800 to 1 : 900,000, classical homocystinuria is biochemically detected by markedly high homocysteine and methionine levels in plasma and urine, with clinical presentation involving mental impairment, vascular complications, dislocated lenses, and skeletal abnormalities [31]. Notably, elevated homocysteine levels are associated with oxidative stress conditions, well known to contribute to the onset and progress of a broad spectrum of diseases. Thus far, besides dietary methionine restriction, the major therapeutic approach for classical homocystinuria consists of administration of pyridoxine (vitamin B6), a precursor of the PLP cofactor [31], although a significant part of patients (approximately half) do not respond to this treatment [32]. The vast majority of the mutations identified in patients with classical homocystinuria are missense mutations resulting in single amino acid substitutions. Whereas most mutations affect the enzyme folding and/or activity [15, 28, 33–39], some of them have been shown to affect enzyme regulation by AdoMet, pointing to such dysregulation as a new pathogenic mechanism in classical homocystinuria [40]. The fact that several variants have impaired activity due to protein misfolding is underlined by the demonstration that some of them are amenable to be functionally rescued by chemical chaperones [34, 36, 38, 39, 41, 42]. A novel therapeutic approach is currently under development based on enzyme replacement therapy using PEGylated recombinant CBS, which has been shown to afford a marked decrease in circulating homocysteine in a mouse model of homocystinuria [43]. This therapeutic approach might be particularly relevant for PLP-unresponsive patients.

The 146 C>T transition in exon 1 of the* CBS* gene generates the clinically relevant p.P49L variant, identified in patients with classical homocystinuria [44–46]. The mutation results in mild to moderate symptoms and sporadic responsiveness to vitamin B6 treatment. When assayed in cell extracts or after purification, the protein variant shows impaired or wild type- (WT-) like canonical activity in the absence or presence of PLP in the assays, respectively, and milder to normal responsiveness to AdoMet [36, 37, 39, 40]. These findings point to defects in PLP incorporation, although the protein variant as purified after recombinant expression in* Escherichia coli* in the presence of suitable chemical chaperones at optimal concentrations exhibits unaffected PLP and heme incorporation, and unperturbed circular dichroism (CD) or UV-visible absorption spectra in the oxidized state [39].

Herein we demonstrate that the p.P49L variant of human CBS displays H_2_S-synthesizing activity largely sensitive to PLP supplementation along the protein purification. The crystallographic structure of a truncated version of CBS p.P49L, devoid of the C-terminal AdoMet binding domain, reveals no major differences at the level of the PLP catalytic site with respect to the WT but slightly increased protein flexibility in the heme surroundings. As a novel finding we report a markedly increased CO affinity of p.P49L as compared to the wild type enzyme,* en route* to enzyme inactivation. The obtained functional and structural data are discussed in light of the proposal that in pathogenic variants of human CBS increased reactivity towards exogenous ligands, such as CO, represents a further molecular mechanism at the basis of classical homocystinuria.

## 2. Materials and Methods

### 2.1. Protein Expression and Purification

Recombinant full-length human CBS p.P49L was expressed and purified as previously described for WT CBS [19] in either the absence or presence of 20 *µ*M PLP, using the herein named pET28b-CBS-p.P49L vector generated in [40]. With this vector as template, site-directed mutagenesis was employed to obtain also a truncated form of the protein (denoted by CBSΔ_409–551_ p.P49L) devoid of the C-terminal 143 residues corresponding to the AdoMet-binding domain. The 1227G>A mutant (cDNA numbering) carrying a premature stop codon at position 409 was generated from pET28b-CBS-p.P49L using the XL Quick Change Kit (Agilent) and the primers 5′-GAAGAAGCCCTGGTGATGGCACCTCCGTG (forward) and 5′-CACGGAGGTGCCATCACCAGGGCTTCTTC (reverse). All vectors were checked for the correct mutation by DNA sequencing. Expression and purification of CBSΔ_409–551_ p.P49L were carried out as described in [20].

Purity of the isolated proteins was assessed by SDS-PAGE and their concentration was determined by the Bradford method [47], whereas the heme concentration in the isolated oxidized proteins was determined using *ε*_428 nm_ = 92,700 M^−1^ cm^−1^ [48].

Unless otherwise stated, the experiments were carried out in 50 mM KPi buffer, 300 mM KCl, 10% glycerol, 100 *µ*M EDTA, pH 7.0 (buffer A).

### 2.2. H_2_S Synthesis Assays

H_2_S production by recombinant human CBS variants was measured at 37°C, either by amperometry using a H_2_S-selective electrode (World Precision Instruments) or by the lead acetate method [5]. Purified CBS (0.5–1 *μ*M) was incubated for 10 minutes with 50 *μ*M PLP, 260 U/ml catalase, and 0.4–2.0 mM homocysteine in the absence or presence of 0.5 mM AdoMet, after which 10 mM cysteine was added to trigger the reaction. Amperometric assays were performed using an ISO-H2S-2 hydrogen sulfide sensor coupled to an Apollo 4000 Free Radical Analyzer (World Precision Instruments). After recording H_2_S production for 3 minutes, the electrode was internally calibrated by adding 4 *μ*M NaHS (corresponding to 2 *μ*M H_2_S at pH 7.0). Finally, 50 *μ*M* o*-acetylserine and 200 nM* Entamoeba histolytica o*-acetylserine sulfhydrylase were added to the reaction mixture to remove H_2_S from solution and bring the signal back to baseline [49]. Activity assays by the lead acetate method were carried out in a thermostated cuvette under stirring, according to [5]. Lead acetate (400 *μ*M) was added to the reaction mix prior to cysteine addition and H_2_S production monitored at 390 nm in an Agilent Cary-60 spectrophotometer.

### 2.3. CO Titrations

UV-visible absorption spectra of oxidized and reduced CBS p.P49L and WT were recorded in an Agilent Cary-60 spectrophotometer. Anaerobic titrations of reduced CBS p.P49L with CO were performed at 20°C in an Agilent Cary-60 or a Shimadzu UVPC-1800 spectrophotometer. Gas exchange was prevented either by filling the quartz cuvette and sealing it with a rubber-cap or by adding mineral oil on top of the aqueous medium. Anaerobic conditions were ensured by nitrogen flushing and addition of glucose oxidase (4 units·ml^−1^), catalase (13 *μ*g·ml^−1^), superoxide dismutase (12 units·ml^−1^), and glucose (3 mM) to scavenge contaminant oxygen, hydrogen peroxide, and superoxide anion. CBS p.P49L and WT (1.4–1.6 *μ*M in heme) were reduced with 90 *μ*M sodium dithionite, diluted from a 45 mM stock solution (quantitated using *ε*_314 nm_ = 8,043 M^−1^·cm^−1^ [50]). CO stock solutions were prepared by equilibrating thoroughly degassed buffer A with the pure gas at 1 atm, yielding 1 mM CO at 20°C. After each CO addition with gas-tight Hamilton syringes, the spectral changes were visually inspected in real time and a new addition was immediately made when no more changes were observed.

According to [16, 19, 20, 51], two apparent *K*_*d*_ (*K*_*d*1_ and *K*_*d*2_) were used to satisfactorily fit the CO affinity data. The *K*_*d*1_ and *K*_*d*2_ values were obtained by fitting the data to (1), where *P*_*L*_ is the concentration of CO-bound CBS p.P49L, *P*_*T*_ and *L*_*T*_ are, respectively, the total CBS p.P49L and CO concentrations, and *α*_1_ and *α*_2_ are, respectively, the protein fractions binding CO at higher (*K*_*d*1_) and lower (*K*_*d*2_) affinity.

(1) $$\begin{array}{c} PL=\frac{\alpha_{1}\left[\left(P_{T}+L_{T}+K_{d1}\right)-\sqrt{{\left(P_{T}+L_{T}+K_{d1}\right)}^{2}-4P_{T}L_{T}}\right]+\alpha_{2}\left[\left(P_{T}+L_{T}+K_{d2}\right)-\sqrt{{\left(P_{T}+L_{T}+K_{d2}\right)}^{2}-4P_{T}L_{T}}\right]}{2}. \end{array}$$

### 2.4. Stopped-Flow Measurements

Time-resolved absorption spectroscopy experiments were carried out in a thermostated stopped-flow instrument (DX.17MV, Applied Photophysics), equipped with a photodiode-array (light path, 1 cm). To avoid light-induced artifacts, the intensity of the white-light incident beam was decreased and a filter cutting UV light at *λ* < 360 nm was employed. Absorption spectra were recorded with an acquisition time of 10 ms per spectrum according to a logarithmic time scale. All reactions were carried out at 25°C in buffer A. CBS p.P49L was thoroughly flushed with nitrogen, after which glucose oxidase (4 units·ml^−1^), catalase (13 *μ*g·ml^−1^), superoxide dismutase (12 units·ml^−1^), and glucose (3 mM) were added to scavenge oxygen, hydrogen peroxide, and superoxide anion. The protein was then placed on ice, protected from light to prevent possible damaging photoreactions. When indicated, CBS p.P49L was incubated with AdoMet for ≥10 minutes, prior to reduction with 90 *µ*M sodium dithionite. CO association kinetics were studied by mixing in the stopped-flow apparatus reduced CBS p.P49L, in the absence or presence of AdoMet, with CO solutions and the spectra recorded over time. CO dissociation kinetics were evaluated by mixing the Fe(II)-CO adduct of CBS p.P49L with NO• stock solutions, prepared by equilibrating degassed ultra-pure water with NO• gas at 1 atm, further kept on ice protected from light.

### 2.5. Spectral Data Analysis

CO affinity titrations and CO binding and dissociation kinetic data were analyzed with the software MATLAB (Mathworks). Global fit analysis of spectral data was performed by singular value decomposition analysis combined with curve fitting [52].

### 2.6. Protein Crystallization

Initial crystallization screenings for CBSΔ_409–551_ p.P49L were performed in 96-well plates at 293 K using a Cartesian mini-Bee nanoliter-drop dispensing robot (Genomic Solutions). These screenings allowed for the identification of one hit for CBSΔ_409–551_ p.P49L from the JCSGplus™ screen (Molecular Dimensions): G10 (0.15 M KBr, 30% w/v PEG 2000 MME). Crystals were optimized at the microliter scale using sitting-drop vapor diffusion with a drop composition of 0.5 *µ*l protein solution (27.4 mg·ml^−1^ in buffer A with 20 *µ*M PLP) and 0.5 *µ*l reservoir solution (0.15 M NaBr, 35% PEG 2000 MME) equilibrated against 500 *µ*l precipitant solution in the well. Dark orange colored small needles as well as big rod-shaped crystals appeared after 12 h at 20°C.

### 2.7. Data Processing and Refinement

Cryoprotection conditions for diffraction experiments were achieved by transferring the crystals to a 5 *μ*l drop of 35% (w/v) PEG 2000 MME, 5% (v/v) glycerol, and 0.15 M NaBr. The crystals were flash-cooled by quick plunging into liquid nitrogen. A single crystal was used for data collection under a nitrogen-gas stream (Oxford Cryosystems 700) on beamline ID30A-3 at the ESRF synchrotron (Grenoble, France) using a PILATUS 6 M detector (Dectris) at a wavelength of 0.9677 Å. After indexing and calculation of a data collection strategy using* EDNA* [53], a wedge of 360° of data was collected using a fine-slicing strategy (0.1° rotation per image). The data set was indexed and integrated with* XDS* [54], the space group assignment was performed with* POINTLESS* [55], and scaling was performed with* AIMLESS* [56], all within the* autoPROC* data-processing pipeline [57]. At this stage an *R*_free_-flag set was created corresponding to 5% of the measured reflections of the data set. Crystals belonged to the monoclinic space group* P*1 with unit cell parameters *a* = 86.2 Å, *b* = 86.8 Å, *c* = 97.8 Å, *α* = 102.6°, *β* = 103.1°, and *γ* = 111.2°. Data were truncated at 2.80 Å. Data reduction and refinement statistics are depicted in Table 1. The structure of the CBSΔ_409–551_ p.P49L variant was solved by molecular replacement using PDB entry 1JBQ devoid of any solvent and cofactors as search model using* phaser* [58] within the* PHENIX* software suite of programs [59]. Based on the Matthews coefficient, the search was performed for six molecules. Automated model building was performed using the* AutoBuild* wizard [60], also within* PHENIX*. Initial refinement rounds were carried out with* BUSTER-TNT* [61] using the macro that accounts for missing parts of the model (“-L”). At this point, electron density features attributed to the heme moieties were easily identified. Iterative cycles of manual model building and refinement were carried out with* COOT* [62] and* BUSTER-TNT* until convergence. Validation was performed with* RAMPAGE* [63] and* MolProbity* [64] as implemented in* PHENIX*.

## 3. Results

### 3.1. Hydrogen Sulfide Synthesis by CBS p.P49L

**Figure 1:**
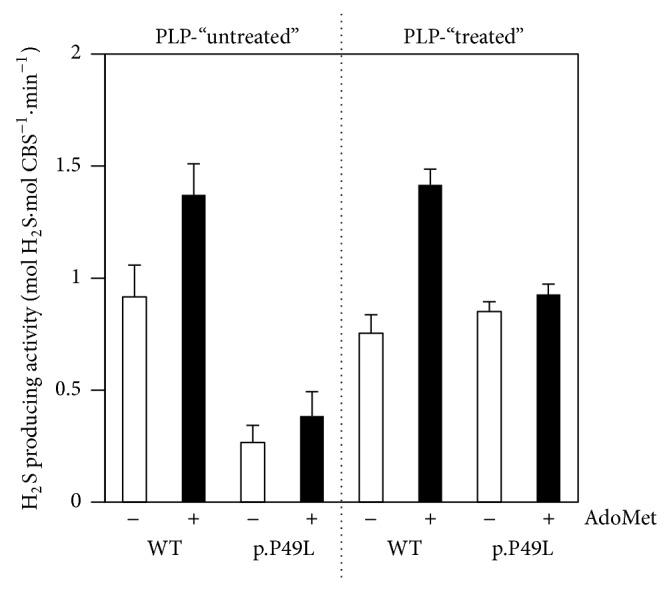
Hydrogen sulfide production by WT and p.P49L CBS. H_2_S producing activity of CBS purified in the absence (PLP-“untreated”) or presence (PLP-“treated”) of PLP (20 *μ*M). *T* = 37°C. Buffer: 50 mM KPi, 300 mM KCl, 10% glycerol, 100 *μ*M EDTA, pH 7.0. Reaction mixture contained 50 *μ*M PLP, 0.4–2.0 mM homocysteine, 260 U/ml catalase, and 10 mM cysteine. Assays were carried out in the absence (−) or presence (+) of 500 *μ*M AdoMet.

H_2_S synthesis by the CBS p.P49L variant using homocysteine and cysteine as substrates was analyzed in comparison with the WT enzyme by amperometric and colorimetric (lead acetate) methods. Two sets of enzyme preparations, purified either in the absence or in the presence of the PLP cofactor, were evaluated in assays run in the presence of PLP. As shown in Figure 1, the p.P49L CBS variant isolated in the absence of PLP (PLP-“untreated”) displays a basal activity more than 3-fold lower than that of the WT enzyme. Despite the markedly impaired enzymatic activity of “untreated” CBS p.P49L, activity stimulation by AdoMet is similar between WT and p.P49L (respectively, 1.5- and 1.4-fold). Conversely, the p.P49L CBS variant purified in the presence of PLP (PLP-“treated”) displays a basal activity similar to the WT enzyme, despite presenting impaired activity stimulation by AdoMet (1.9-fold for WT to be compared with 1.1-fold for p.P49L). The AdoMet activation factor (≤2-fold) observed for the WT enzyme was slightly lower than usually reported (2–5-fold). This could be related to a fraction of the enzyme lacking the C-terminal domain, which was also observed for the CBS p.P49L variant (see Supplementary Figure S1 in the Supplementary Material available online at https://doi.org/10.1155/2017/8940321).

### 3.2. Structure of CBS p.P49L Variant

In an attempt to understand the structural impact of the proline-to-leucine substitution at position 49 of human CBS, we have determined the X-ray structure of a truncated form of the p.P49L variant (PDB entry 5MMS), lacking the C-terminal 143 residues (henceforth designated as CBSΔ_409–551_ p.P49L), similarly to the reported structure of truncated WT CBS (PDB entry 1JBQ) [65]. Crystals belong to the triclinic space group* P*1 with cell dimensions *a* = 86.2, *b* = 86.8, and *c* = 97.8 Å, *α* = 102.6°, *β* = 103.1°, and *γ* = 111.2°.* XDS* as implemented in* autoPROC* clearly identifies two different lattices in the diffraction pattern rotated by 121.4° relative to each other. This diminishes the quality of the overall statistics since in some directions an almost perfect superposition of reflections makes the integration difficult (data collection and refinement statistics are depicted in Table 1).

**Figure 2:**
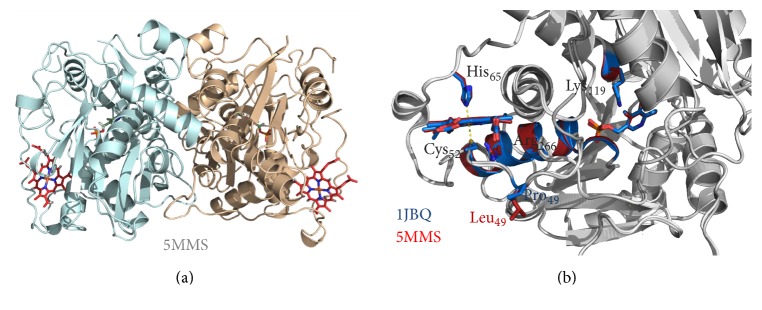
Structure of CBS p.P49L. X-ray crystallographic structure of CBSΔ_409–551_ p.P49L solved at 2.80 Å resolution (PDB entry 5MMS). (a) Cartoon representation of the protein dimer, each monomer being represented in a different color. Pyridoxal 5′-phosphate (PLP) and heme moieties shown in stick representation. (b) Structure superposition of CBSΔ_409–551_ p.P49L (PDB entry 5MMS) and truncated WT CBS (PDB entry 1JBQ), both colored in grey except for most relevant regions and residues, where CBSΔ_409–551_ p.P49L is colored in red and CBSΔ_409–551_ WT in blue; zoom in on the PLP and heme moieties, highlighting the proline-to-leucine substitution, as well as the R266 residue and *α*-helix 8 proposed to mediate communication between the heme and the PLP active site. Figure generated with PyMOL 1.8.2 (The PyMOL Molecular Graphics System, Version 1.8 Schrödinger, LLC).

There are six molecules in the asymmetric unit corresponding to a Matthews coefficient [66] of 2.31 Å^3^·Da^−1^ and a solvent content of approximately 47%. The structure was refined to 2.80 Å resolution with *R*_cryst_ of 18.2% and *R*_free_ of 22.1%. The final model comprises the residues from R45 to E400 (in chain D), 6 hemes, 6 PLP molecules, 3 sodium ions, and 185 water molecules. The hemes are axially bridged by C52 and H65 and the PLP moieties covalently linked to the polypeptide chain through K119. The maps are generally of good quality except for two disordered loops (T193 to S199 and Q295 to T300), for which only in chain D there were complete electron densities. This contrasts with the published structure of the truncated human CBS WT (PDB entry 1JBQ), where the T193-S199 loop could not be modeled. The variant dimeric structure, shown in Figure 2(a), displays an essentially identical overall fold with respect to the WT enzyme (r.m.s.d. of 0.4 Å for 344 aligned C_*α*_ carbon atoms between chain D of CBSΔ_409–551_ p.P49L and chain A of 1JBQ) and highly conserved features in the PLP active site and the N-terminally located heme moiety (Figure 2(b)).

### 3.3. Spectral Properties of Ferrous p.P49L CBS

**Figure 3:**
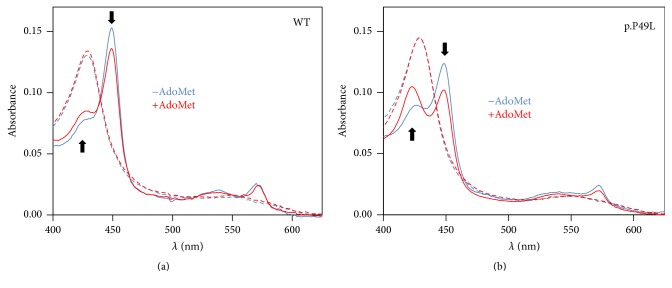
Absorption spectra of WT and p.P49L CBS. Absorption spectra of WT (a) and p.P49L (b) CBS (1.4–1.6 *µ*M in heme) recorded at 20°C, in degassed buffer A (50 mM KPi, 300 mM KCl, 10% glycerol, 100 *μ*M EDTA, pH 7.0), containing glucose oxidase (4 units·ml^−1^), catalase (13 *μ*g·ml^−1^), superoxide dismutase (12 units·ml^−1^), and glucose (3 mM). Spectra were collected in the oxidized state (dashed lines) and upon protein reduction (solid lines) by addition of 90 *μ*M sodium dithionite, in the absence (blue lines) and presence (red lines) of AdoMet (500 *μ*M). Arrows highlight direction of the spectral changes caused by AdoMet in the reduced proteins.

In the absence of clear structural clues for pathogenicity of the p.P49L mutation, we sought to evaluate by UV-visible absorption spectroscopy the impact of this residue substitution on the protein redox spectra, largely dominated by the heme absorption. In WT CBS, heme reduction leads to notable changes in the protein absorption spectrum, with a shift of the Soret band from 428 nm to 449 nm (Figure 3(a)). Upon incubation of the enzyme with AdoMet prior to reduction, the spectrum of the reduced enzyme is affected by a decrease in the 449 nm band intensity and the appearance of a feature centered at ~424 nm (Figure 3(a), red solid line). Regardless of AdoMet, in the oxidized state, p.P49L CBS exhibits no differences in the absorption spectrum as compared to the WT protein (Figure 3, dashed lines). In contrast, major differences can be noted by comparing the spectra of the two proteins in the reduced state, with the mutant displaying markedly more pronounced appearance of the 424 nm spectral feature and decrease of the 449 nm band (Figure 3(b), blue solid line), both further elicited in the AdoMet-bound protein (Figure 3(b), red solid line).

### 3.4. Enhanced Affinity of p.P49L CBS for CO

**Figure 4:**
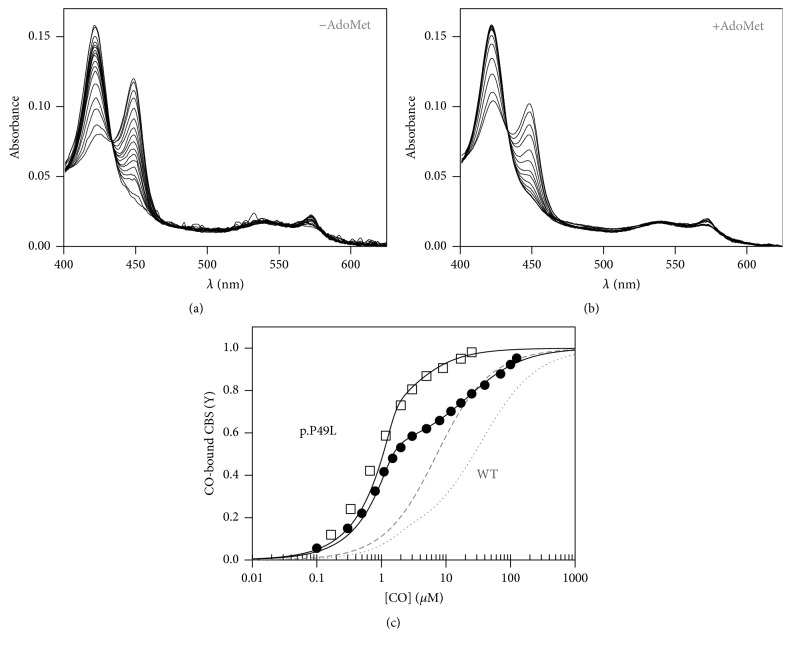
Enhanced CO affinity of p.P49L CBS. Absorption spectra collected upon anaerobic titration of reduced CBS p.P49L (1.4–1.6 *μ*M in heme) with CO, in the absence (a) or presence (b) of AdoMet. *T* = 20°C. (c) Titration profiles obtained by global fit of the spectral data acquired in the absence (full circles) or presence (hollow squares) of 500 *μ*M AdoMet. Data were best fitted according to (1), yielding *K*_*d*CO,1_ = 0.05 *μ*M (60%) and *K*_*d*CO,2_ = 22.0 *μ*M (40%) for AdoMet-free CBS p.P49L and *K*_*d*CO,1_ ≤ 0.03 *μ*M (70%) and *K*_*d*CO,2_ = 2.1 *μ*M (30%) for the AdoMet-bound enzyme. Gray lines represent titration curves for WT CBS in the absence (dotted line) and presence (dashed line) of 500 *μ*M AdoMet.

Prompted by the observed spectral differences between reduced p.P49L and WT CBS, we analyzed the affinity of the mutated protein for the physiologically relevant CO ligand by performing anaerobic CO titrations. Similarly to the WT protein, conversion of ferrous CBS p.P49L to the CO adduct resulted in the appearance of a band centered at 422 nm (Figures 4(a) and 4(b)). Global fit analysis of the spectral data set acquired along the titration revealed a much higher CO affinity (>50-fold) of p.P49L CBS (Figure 4(c), full circles) as compared to the WT enzyme (Figure 4(c), dotted line). Notably, as previously reported by Vicente et al. [20] for WT CBS (Figure 4(c), dashed line) and consistent with the effect of AdoMet on the spectrum of reduced p.P49L CBS (Figure 3(b)), preincubation of the protein variant with AdoMet further enhances the CO affinity (Figure 4(c), hollow squares). CO titrations allowed us to estimate the following *K*_*d*_ values: *K*_*d*CO,1_ = 0.06 ± 0.03 *μ*M and *K*_*d*CO,2_ = 21 ± 5 *μ*M (with 60% and 40% relative amplitude, respectively) for AdoMet-free p.P49L CBS and *K*_*d*CO,1_ ≤ 0.03 *μ*M and *K*_*d*CO,2_ = 1.5 ± 0.6 *μ*M (with 70% and 30% relative amplitude, respectively) for the AdoMet-bound enzyme. It should be noted that the CO affinity of the AdoMet-bound p.P49L variant is so high that the *K*_*d*CO,1_ value actually represents an upper limit. A possible direct interference of CO with AdoMet was ruled out by performing a control experiment where an AdoMet solution was equilibrated with CO gas (or N_2_ as control), yielding no spectral changes (*not shown*).

### 3.5. Kinetics of CO Binding and Dissociation from p.P49L CBS

**Figure 5:**
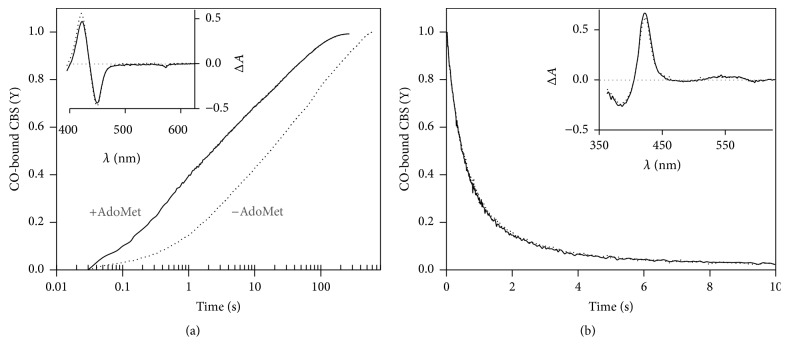
Kinetics of CO binding to ferrous CBS p.P49L. (a) Reaction time courses measured in the absence (dotted line) or presence of AdoMet (500 *μ*M before mixing; solid line). Spectral data collected after stopped-flow mixing 1 mM CO with reduced CBS p.P49L (1.5 *μ*M in heme) at 25°C, in 50 mM potassium phosphate, 300 mM KCl, 10% glycerol, pH 7.0, containing 2 mM glucose, 4 units·ml^−1^ glucose oxidase, 13 *μ*g·ml^−1^ catalase, and 6 units·ml^−1^ superoxide dismutase. Fitted rate constants (% reaction amplitude):* k*_1_ = 0.48 s^−1^ (25%),* k*_2_ = 0.05 s^−1^ (30%), and* k*_3_ = 0.006 s^−1^ (45%) for AdoMet-free CBS p.P49L (*t*_1/2_ = 19.5 s) and* k*_1_ = 2.55 s^−1^ (35%),* k*_2_ = 0.26 s^−1^ (30%), and* k*_3_ = 0.022 s^−1^ (35%) for the AdoMet-bound enzyme (*t*_1/2_ = 2.3 s).* Inset,* optical transitions obtained by global fit analysis of the spectral data acquired in the absence (dotted line) or presence of AdoMet (solid line). (b) Time courses of CO displacement from ferrous CBS p.P49L by 900 *μ*M NO•, acquired in the absence (dotted line) or presence of AdoMet (solid line; 500 *μ*M before mixing). Traces were best fitted with the following rate constants (% reaction amplitude):* k*_1_ = 1.93 s^−1^ (75%) and* k*_2_ = 0.035 s^−1^ (25%) for AdoMet-free CBS p.P49L;* k*_1_ = 1.96 s^−1^ (75%) and* k*_2_ = 0.038 s^−1^ (25%) for the AdoMet-bound enzyme.* Inset*, optical transitions obtained by global fit analysis of the spectral data acquired in the absence (dotted line) or presence of AdoMet (solid line).

The markedly higher CO affinity of p.P49L CBS as compared to the WT enzyme led us to investigate by time-resolved absorption spectroscopy the kinetics of CO binding to and dissociation from ferrous p.P49L CBS (Figure 5). Upon stopped-flow mixing reduced CBS p.P49L with 1 mM CO, the observed spectral changes were identical in shape to those shown for the CO titrations in Figure 4, that is, the predominant 449 nm Soret band and the 424 nm spectral feature both shifted to 422 nm, with similar optical transitions for the AdoMet-free and AdoMet-bound CBS p.P49L (inset to Figure 5(a)). Global fit analysis of the kinetic data revealed that both AdoMet-free and AdoMet-bound p.P49L react with CO according to multiphasic time courses (Figure 5(a)), as previously shown for WT CBS [19, 20, 51, 67]. Interestingly, despite the markedly increased CO affinity of p.P49L with respect to the WT protein, under identical experimental conditions CO binding to the either of the two proteins in the absence of AdoMet proceeds at comparable rates (*t*_1/2_ = 26.2 ± 9.6 s for CBS p.P49L, Figure 5(a), to be compared with *t*_1/2_ = 34.5 ± 10.5 s of WT, not shown). In the presence of AdoMet, where the spectral changes (Figure 3) and the enhanced CO affinity (Figure 4) of CBS p.P49L point to significant effects on the heme properties, a marked increase in CO association rates is observed, with* t*_1/2_ decreasing to 5.4 ± 3.1 s (Figure 5(a)), to be compared with* t*_1/2_ = 17.0 ± 2.7 s for WT CBS (not shown).

The kinetics of CO dissociation from CBS p.P49L was evaluated by anaerobically mixing in the stopped-flow apparatus the CO-bound ferrous protein with authentic NO• (900 *µ*M after mixing) and monitoring the conversion of the 422 nm hexacoordinate CO-bound adduct spectrum into that of the pentacoordinate NO•-bound adduct with a broad absorption band centered at 395 nm (inset to Figure 5(b)). Global fit analysis of the kinetic data revealed that under identical experimental conditions CO is displaced by NO• in CBS p.P49L at comparable rates (*k*_1_ = 2.0 ± 0.1 s^−1^ and* k*_2_ = 0.37 ± 0.05 s^−1^, with 75% and 25% relative amplitude, respectively) to the WT enzyme (*k*_1_ = 2.0 ± 0.1 s^−1^ and* k*_2_ = 0.33 ± 0.03 s^−1^, with 75% and 25% relative amplitude, respectively;* data not shown*), showing no effect of AdoMet (Figure 5(b)).

## 4. Discussion

Classical homocystinuria is an inborn error of metabolism associated with deficiency in cystathionine *β*-synthase (CBS), a key enzyme in the transsulfuration pathway of methionine metabolism. By catalyzing the conversion of homocysteine and serine into cystathionine, the enzyme prevents an excessive increase in homocysteine levels, a pathological condition associated with oxidative stress and clinical complications in the vascular, neurological, and skeletal systems. CBS also has a relevant role in human physiology by being a major source of H_2_S, a key endogenous signaling molecule whose dysregulation is at the basis of several human pathologies, from cardiovascular and neurodegenerative diseases to cancer. Thus far, despite decades of research on classical homocystinuria, a full understanding of the molecular events at the basis of the pathogenicity of several CBS mutations remains elusive, although protein misfolding, dysfunctional regulation by AdoMet, and impaired enzymatic activity have been put forward for some mutations [15, 28, 33–39].

This prompted us to investigate in the present study a reportedly mild pathogenic mutation associated with classical homocystinuria, a proline-to-leucine substitution at residue 49 in human CBS [44–46]. The recombinant CBS p.P49L variant was expressed in* E. coli*, purified, and characterized both structurally and functionally. In line with previous reports focused on the canonical cystathionine synthase activity, this variant displayed a H_2_S synthesizing activity remarkably sensitive to PLP supplementation along the purification procedure [36, 37, 39, 40]. The functional rescue of p.P49L H_2_S synthesis by PLP is indicative of a decreased affinity of this variant for the cofactor, a frequently observed consequence of missense mutations potentially associated with protein misfolding. The functional recovery of p.P49L observed upon PLP supplementation during protein purification was however not fully matched in terms of activity stimulation by AdoMet, as the H_2_S-synthesizing activity of p.P49L showed poor responsiveness to AdoMet (Figure 1) as compared to its cystathionine synthase activity [36, 37, 39, 40]. This is not surprising since for other CBS variants it has been shown that the extent of the stimulatory effect of AdoMet can differ between the canonical cystathionine synthase and the H_2_S-synthesizing activities [28].

Further attempting to understand the molecular basis of pathogenicity of this mutation, the crystallographic structure of a truncated form of the CBS p.P49L variant (CBSΔ_409–551_ p.P49L) was obtained at 2.8 Å resolution and compared with that of the truncated WT enzyme (PDB 1JBQ) [65]. Within the obtained resolutions, the structures display highly conserved features (Figure 2), particularly inspecting the PLP binding pocket with the cofactor covalently bound to K119, the heme ligands C52 and H65, *α*-helix 8 (responsible for the heme-PLP communication), the R266 residue forming a salt-bridge with C52, and the flexible loop where the mutated P49 residue is located. Therefore, at first glance, the structural data do not seem to provide a clue for the pathogenicity of the mutation.

The effect of the mutation on the spectroscopic and ligand-binding properties of the heme moiety was also investigated. The first hint for a perturbation in the CBS p.P49L heme microenvironment arose from inspection of the dithionite-reduced spectrum of this protein variant (Figure 3). Indeed, in the spectrum of reduced CBS p.P49L, the dominating 449 nm band assigned to the hexacoordinate ferrous heme with C52 and H65 as axial ligands shows a significant intensity decrease as compared to the WT enzyme and the appearance of a band at 424 nm (Figure 3(b)). The latter spectral feature has been assigned to a ligand exchange process in CBS leading to formation of an enzymatic species (called C-424), in which the cysteine thiolate ligand is replaced by a neutral species [14, 15], negatively impacting the enzymatic activity. In WT CBS, this ligand exchange process occurs very slowly (>48 h at 37°C, [14]) in the presence of excess reductant. Similarly to p.P49L, other CBS variants have been previously reported to display an increased propensity to form the C-424 species, particularly CBS variants with mutated residues in *α*-helix 8 [28]. Furthermore, as observed for the WT CBS (Figure 3(a)), incubating the p.P49L variant with AdoMet prior to reduction further enhances the conversion of the “normal” 449 nm into the ligand-exchanged C-424 species in the reduced protein (Figure 3(b)). Interestingly, among the several CBS variants studied by Yadav and coworkers [28], p.T257V shows the most similar spectra to CBS p.P49L and, like this variant, it displays WT-like (and PLP-dependent) H_2_S-generating activity and impaired activation by AdoMet [28]. Altogether, the spectral data herein reported point to changes at the heme moiety of CBS p.P49L that were further explored by evaluating the CO binding properties of the protein variant. CBS has been shown to be inhibited in vitro by exogenous ligands like CO and NO• [16–20], with different lines of evidence pointing to a physiological relevance of this regulatory mechanism in vivo [21–27] (see below).

By performing CO titrations under anaerobic conditions, we observed spectral changes (Figure 4) consistent with the formation of the hexacoordinate ferrous-CO adduct, with the C52 thiolate or the yet unknown “X” ligand of the C-424 species being replaced by CO, and the heme retaining the H65 endogenous ligand. Notably, the CO affinity, herein measured for the first time in a CBS variant, is markedly increased (≥50-fold) in CBS p.P49L with respect to the WT enzyme (Figure 4(c), [16, 20, 51]). As previously described for WT CBS, the CO titrations followed a biphasic profile, which has been previously attributed to heterogeneity in the heme microenvironment [16] or anticooperativity between hemes within a CBS dimer [51]. The remarkably higher CO affinity of CBS p.P49L is essentially due to the extremely low *K*_*d*CO,1_ (0.06 ± 0.03 *μ*M), close to the detection limit of the experimental setup. Notably, and as previously observed for the WT enzyme [20], incubation of CBS p.P49L with AdoMet induced a further increase in CO affinity (Figure 4(c)) and, therefore, only an upper limit value for *K*_*d*CO,1_ (≤0.03 *μ*M) could be estimated. In the WT enzyme, the increased CO affinity observed in the presence of AdoMet is fully matched with an enhanced propensity for CO inhibition of the protein H_2_S producing activity [20]. Based on the remarkable increase in CO affinity herein documented for CBS p.P49L, this protein variant is expected to be more prone to inhibition at low physiological CO levels. This may represent a more general mechanism of pathogenesis in classical homocystinuria, if other pathogenic CBS mutations will be demonstrated to lead to enhanced CO affinity, as shown for CBS p.P49L in the present study.

Although direct evidence for ferrous CBS formation in vivo is still missing in the literature, several reports have attested the physiological role of CBS inhibition by CO (reviewed in [26, 27]), which requires the heme to be in the ferrous state. Regarding regulation of cerebral microcirculation by hypoxia, decreased oxygen levels impair CO production by heme oxygenase HO-2 and the release of CBS inhibition by CO promotes H_2_S synthesis that in turn mediates vasodilation of precapillary arterioles [23]. Stress-inducible levels of CO in mice liver cause metabolomic changes consistent with CBS inhibition, decrease in hepatic H_2_S, and concomitant stimulation of HCO_3_^−^-dependent bile output in wild type, but not in heterozygous CBS knockout mice [22]. Another proposed mechanism concerns the CO-mediated regulation of glucose utilization, where CBS inhibition by CO drives the demethylation of phosphofructokinase/fructose bisphosphatase type-3 (PFKFB3), diverting glucose from the glycolytic towards the NADPH-generating pentose phosphate pathway, with implications in chemoresistance and oxidative stress resistance in cancer cells [24]. Moreover, Kabil et al. [25] have recently shown that, under endoplasmic reticulum stress conditions, CBS inhibition by CO, combined with CSE induction, flips the CSE substrate preference from cystathionine to cysteine, transiently stimulating H_2_S production. These multiple lines of evidence provide compelling though still indirect evidence for the formation of ferrous CBS in vivo. In line with these observations, an NADPH-dependent diflavin enzyme, methionine synthase reductase, has been shown to reduce the CBS heme in vitro in the presence of CO or nitrite, generating, respectively, the ferrous-CO or ferrous-NO CBS adducts [18, 67, 68]. In light of this evidence, the high affinity of CBS p.P49L for CO is fully compatible with the formation of the ferrous-CO adduct at physiological CO concentrations.

To gain mechanistic insight into this high CO affinity, we studied by stopped-flow absorption spectroscopy the kinetics of CO association/dissociation to/from the reduced heme of this protein variant (Figure 5). Similarly to the WT [19, 20, 51], CO binding to reduced CBS p.P49L followed a multiphasic time-course (Figure 5(a)). Taking into account the markedly increased CO affinity of this protein variant, surprisingly the reaction proceeded only slightly faster (*t*_1/2_ = 26.2 ± 9.6 s) than for the WT enzyme (*t*_1/2_ = 34.5 ± 10.5 s) under identical experimental conditions. Despite this minor difference, in the presence of AdoMet the fold increase in the CO association rate was overall higher in the p.P49L variant (~4.5-fold) than in the WT enzyme (~2-fold) under identical experimental conditions. Furthermore, by analyzing the kinetics of CO replacement by NO• in this protein variant, we observed essentially identical kinetic traces for the AdoMet-free and AdoMet-bound CBS p.P49L (Figure 5(b)) and for the WT enzyme under the same experimental conditions (*not shown*).

**Scheme 1:**
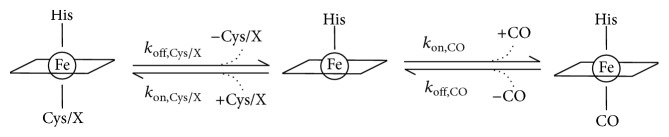


The kinetics of CO association and dissociation therefore do not provide a clear cut explanation for the markedly higher CO affinity of CBS p.P49L, which requires further inspection. In Scheme 1 are represented the reaction steps for conversion of the hexacoordinate ferrous CBS, with the heme Fe ligated to H65 and either C52 or the unknown “X” ligand in the C-424 species, into the ferrous-CO adduct. It has been previously postulated for the WT enzyme that CO association to the ferrous CBS heme is rate-limited by dissociation of C52 [19, 20, 51]. Since we observed similar CO association kinetics for WT and p.P49L, where a fraction of the mutant enzyme is likely to be in the ligand-exchanged C-424 state, the CO association appears to be limited by the off-rate of the endogenous ligand regardless of its nature, C52 or “X” (*k*_off,Cys/X (p.P49L)_ ≈ *k*_off,Cys/X (WT)_ in Scheme 1). Taking into account the fact that the kinetics of CO dissociation were almost identical for WT and p.P49L CBS, regardless of AdoMet being present (*k*_off,CO (p.P49L)_ ≈ *k*_off,CO (WT)_ in Scheme 1), the dramatic increase in CO affinity of the p.P49L variant compared to the WT should be related to a slower rebinding of the endogenous ligand, C52 or “X” (*k*_on,Cys/X (p.P49L)_ ≪ *k*_on,Cys/X (WT)_ in Scheme 1), and/or a faster combination of CO with the transiently generated pentacoordinate species (*k*_on,CO (p.P49L)_ ≫ *k*_on,CO (WT)_ in Scheme 1).

**Figure 6:**
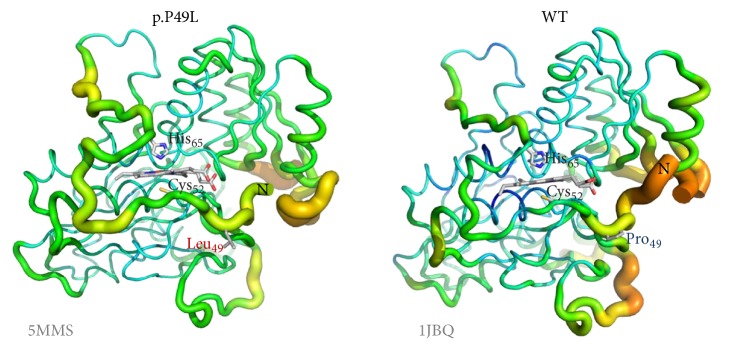
Increased flexibility of the heme binding loop in p.P49L CBS. Representation of *B* factor variation along the structure of CBS p.P49L (PDB entry 5MMS) and WT (PDB entry 1JBQ) monomer, displaying in first plane the regions encompassing the proline-to-leucine mutation (sticks) and the heme ligands C52 and H65. Flexibility can be visualized both by the thickness of the structural element and the respective color: highest flexibility represented in red (hot) thick elements; lowest flexibility in thin blue (cold) elements (color scale: red > orange > yellow > green > blue). Figure generated with PyMOL 1.8.2 (The PyMOL Molecular Graphics System, Version 1.8 Schrödinger, LLC).

Regardless of these mechanistic details, the perturbed spectrum of the reduced protein and its remarkably higher affinity for CO point to possibly subtle structural changes in the CBS p.P49L variant affecting heme reactivity. To this end, we further compared the structures of the variant and WT enzymes in terms of local flexibility evaluated based on the B factor (Figure 6). This analysis interestingly reveals that the differences in flexibility are mostly located in specific regions of the protein. The *α*-helix 8, where some mutations have been shown to affect the heme spectral properties and the H_2_S-generating activity similarly to p.P49L, displays comparable rigidity between CBS p.P49L and the WT enzyme. We thus looked in greater detail at the heme binding region (Figure 6) and found in CBS p.P49L an increased flexibility of the loop surrounding the C52 ligand, which expands to the regions between the C52 and H65 ligands and even after the latter residue. The p.P49L structure therefore displays a higher flexibility in the region harboring both heme ligands, which provides a possible structural basis for the proposed slower rebinding of the endogenous C52/X ligands upon CO dissociation, thereby accounting for the increased affinity of CBS p.P49L for CO.

## 5. Conclusions

Cystathionine *β*-synthase (CBS) is a key enzyme in the transsulfuration pathway that prevents oxidative stress conditions, both controlling homocysteine levels and promoting the expression of antioxidant enzymes through the synthesis of H_2_S. Being implicated in metabolic, oncologic, and neurodegenerative diseases, CBS is currently recognized as a promising drug target. Mutations in the* CBS* gene can lead to classical homocystinuria, a human disease associated with oxidative stress that affects the vascular, neurological, and skeletal systems. Protein misfolding, enhanced propensity to aggregation, decreased cofactor affinity, and dysfunctional regulation by the allosteric activator AdoMet, together with impaired enzymatic activity, have been proposed to account for the pathogenicity of several CBS mutations. As a novel finding, herein we reported that a clinically relevant variant of CBS (p.P49L) has markedly increased affinity for CO, a known inhibitor of CBS. On this basis, this variant is expected to be much more prone than WT CBS to be inactivated by CO at the physiological levels occurring in vivo, thereby contributing to pathogenicity. The enhanced affinity for inhibitory gaseous ligands documented here may represent a new pathogenic mechanism at the basis of CBS-related diseases, like classical homocystinuria.

## Supplementary Material

SDS-PAGE analysis of CBS preparations

## Figures and Tables

**Table 1:** Data reduction and refinement statistics of CBS p.P49L structure.

	p.P49L CBS variant
PDB entry	5MMS
*Data collection*	
Synchrotron	ESRF (Grenoble, France)
Beamline	ID30A-3
Wavelength (Å)	0.968
Space group	*P*1
Unit cell	
*a*, *b*, *c* (Å)	86.2, 86.8, 97.8
*α*, *β*, *γ* (°)	102.7, 103.1, 111.2
Resolution range^a^ (Å)	76.35–2.80 (2.90–2.80)
Total number of reflections	121227 (1141)
Number of unique reflections	58862 (571)
Completeness (%)	98.5 (95.6)
Multiplicity	2.1 (2.0)
〈*I*/*σ*(*I*)〉	4.8 (1.5)
*R*_meas_^b^ (%)	17.4 (68.7)
*R*_pim_^c^ (%)	11.2 (45.1)
*CC*_1/2_^d^ (%)	97.6 (62.1)
Wilson *B*-factor (Å^2^)	41.8

*Refinement*	
*R*_cryst_^e^ (%)	18.2 (27.2)
*R*_free_^f^ (%)	22.1 (33.1)
Number of non-H atoms	16452
Protein	15916
Ligands	351
Waters	185
r.m.s.d bonds (Å)	0.010
r.m.s.d. angles (°)	1.12
Protein residues	2077
Ramachandran plot	
Most favoured (%)	96.6
Allowed (%)	3.2
Outliers (%)	0.3
Rotamer outliers (%)	0.3
Clashscore	0.46
*MolProbity* score^g^	0.88
*B*-factors (Å^2^)	45.4
Protein	41.3
Ligands	29.7

^a^Information in parenthesis refers to the last resolution shell. ^b^*R*_meas_ = Σ_*hkl*_(*n*/*n* − 1)^1/2^Σ_*i*_ | *I*_*hkl*,*j*_ − 〈*I*_*hkl*,*j*_〉 | /Σ_*hkl*_Σ_*j*_*I*_*hkI*,*j*_, where *n*_*h*_ denotes multiplicity and *j* the*j*-th reflection *hkl*. ^c^*R*_pim_ = Σ_*hkl*_(1/*n* − 1)^1/2^Σ_*i*_ | *I*_*hkl*,*j*_ − 〈*Ihkl*, *j*〉 | /Σ_*hkl*_Σ_*i*_*I*_*hkI*,*j*_, where *n*_*h*_ denotes multiplicity and *j* the *j*-th reflection *hkl*. ^d^*CC*_1/2_ is as described previously [69]. ^e^*R*_cryst_ = ∑_*hkl*_‖*F*_obs(*hkl*)_ | −|*F*_calc(*hkl*)_‖/∑_*hkl*_|*F*_obs(*hkl*)_|, where *F*_obs(*hkl*)_ and *F*_calc(*hkl*)_ are the observed and calculated structure factors for reflection (*hkl*), respectively. ^f^*R*_free_ was calculated as *R*_cryst_ but using only 5% of reflections randomly selected and omitted from refinement. ^g^*MolProbity* score provides a single number that represents the central *MolProbity* protein quality statistics; it is a log-weighted combination of Clashscore, Ramachandran not favored, and bad side-chain rotamers, giving one number that reflects the crystallographic resolution at which those values would be expected.

## Abbreviations

AdoMet: s-Adenosyl-l-methionine
CBS: Cystathionine *β*-synthase
CBSΔ_409–551_: Truncated cystathionine *β*-synthase lacking the C-terminal 143 residues
CBS p.P49L: Cystathionine *β*-synthase variant harboring a proline-to-leucine substitution at residue 49
CO: Carbon monoxide
CSE: Cystathionine *γ*-lyase
EDTA: Ethylenediaminetetraacetic acid
H_2_S: Hydrogen sulfide
KPi: Potassium phosphate
MME: Monomethyl ether
MST: Mercaptopyruvate sulfurtransferase
NO: Nitric oxide
PEG: Polyethylene glycol
PLP: Pyridoxal 5′-phosphate
r.m.s.d.: Root mean square deviation
SDS-PAGE: Sodium dodecyl sulfate polyacrylamide gel electrophoresis
WT: Wild type.
