# Supplementary material for: A Clinically Relevant Variant of the Human Hydrogen Sulfide-Synthesizing Enzyme Cystathionine β-Synthase: Increased CO Reactivity as a Novel Molecular Mechanism of Pathogenicity?
Source: Oxid Med Cell Longev. 2017 Mar 22;2017:8940321. doi: 10.1155/2017/8940321 (PMC5381205; doi:10.1155/2017/8940321)
Supplement: Supplementary file 1 — SDS-PAGE analysis of CBS preparations [file 8940321.f1.pdf]

SUPPLEMENTAL MATERIAL

A clinically relevant variant of the human hydrogen sulfide-synthesizing enzyme cystathionine β-synthase: increased CO reactivity as a novel molecular mechanism of pathogenicity?

João B. Vicente[1,*], Henrique G. Colaço[2], Francesca Malagrinò[3,4], Paulo E. Santo[1,5], André Gutierres[1], Tiago M. Bandeiras[1,5], Paula Leandro[6], José A. Brito[1], and Alessandro Giuffrè[3,*]

1 - Instituto de Tecnologia Química e Biológica António Xavier, Universidade Nova de Lisboa, Oeiras, Portugal
2 - Instituto Gulbenkian da Ciência, Oeiras, Portugal
3 - CNR Institute of Molecular Biology and Pathology, Rome, Italy
4 - Department of Biochemical Sciences, Sapienza University of Rome, Italy.
5 - Instituto de Biologia Experimental e Tecnológica, Oeiras, Portugal
6 - Research Institute for Medicines and Department of Biochemistry and Human Biology, Faculty of Pharmacy, University of Lisbon, Portugal

Supplementary Figure 1. SDS-PAGE analysis of CBS preparations. Analysis of CBS samples employed in this study, corresponding to full-length CBS WT and p.P49L purified in the absence (‘untreated’) or presence (‘treated’) of PLP (20 µM). Truncated WT and p.P49L CBS lacking the C-terminal AdoMet-binding domain were analyzed for comparison with the full-length protein. Denatured protein samples were loaded onto a pre-cast NuPAGE™ 10% Bis-Tris gel (Invitrogen) and ran at 200 V. Protein bands were stained with InstantBlue™ (Expedeon) according to the manufacturer’s instructions. Protein marker: NZYColour Protein Marker II (Nzytech).

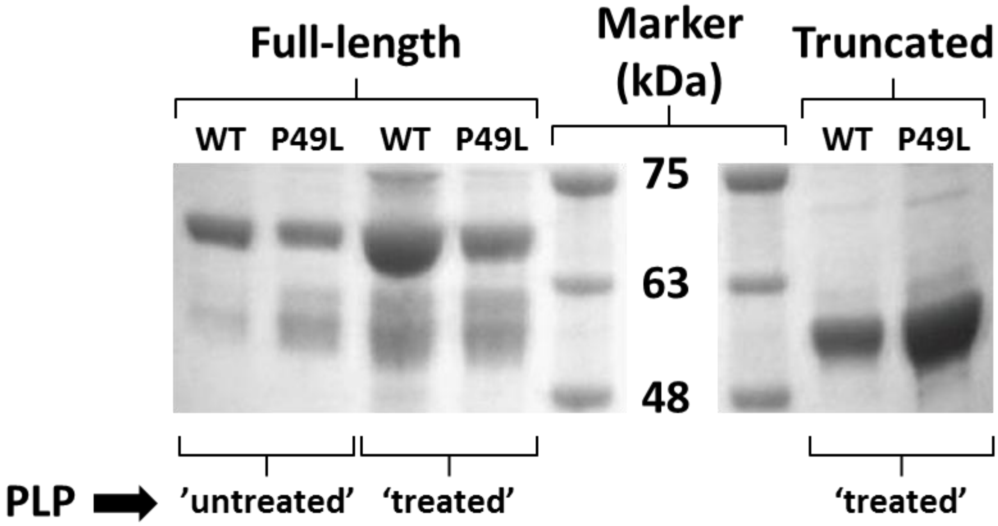

Supplementary Figure 1

Vicente et al (2017)
